# Ischemic Complications After Parent Artery Occlusion for Ruptured Posterior Cerebral Artery Aneurysm With a Fetal-Type Posterior Communicating Artery: A Case Report

**DOI:** 10.7759/cureus.108130

**Published:** 2026-05-02

**Authors:** Takeshi Matsufusa, Kazuhide Maeshima, Nao Otani, Nobuyuki Miyatake, Hideki Takemoto

**Affiliations:** 1 Neurological Surgery, Japanese Red Cross Wakayama Medical Center, Wakayama City, JPN

**Keywords:** endovascular treatment (evt), fetal-type posterior cerebral artery, parent artery occlusion, posterior cerebral artery aneurysm, subarachnoid hemorrhage

## Abstract

Posterior cerebral artery (PCA) aneurysms are uncommon, and many are considered to be dissecting aneurysms. In ruptured cases, parent artery occlusion (PAO) is often selected as a treatment strategy. However, the morphology of the posterior communicating artery (PCoA) may influence the risk of postoperative ischemic complications. We report the case of a 76-year-old man with a sudden-onset headache and impaired consciousness. He was diagnosed with subarachnoid hemorrhage (SAH) and Hunt and Kosnik grade V. Imaging revealed a ruptured aneurysm located in the distal P2 segment of the left PCA. The ipsilateral P1 segment was hypoplastic, and the PCA was mainly supplied via a fetal-type PCoA. To prevent rebleeding, PAO was performed via a PCoA approach. The aneurysm and the proximal PCA segment were embolized with coils. Although complete occlusion was achieved, postoperative magnetic resonance imaging (MRI) demonstrated infarctions in the lateral thalamus and the medial temporal and occipital lobes. These findings were consistent with ischemia in the territories of the thalamogeniculate artery and the temporal branches of the PCA. The patient developed right homonymous hemianopia and higher cortical dysfunction, including attention and memory impairment, and was transferred to a rehabilitation hospital with a modified Rankin Scale (mRS) score of 4. While PAO effectively prevented rebleeding in this case, this report suggests a possible association between fetal-type PCoA anatomy and ischemic complications after PAO. Careful treatment planning based on detailed preoperative vascular anatomical assessment is essential.

## Introduction

Posterior cerebral artery (PCA) aneurysms account for approximately 0.7%-2.2% of all intracranial aneurysms and 7%-15% of aneurysms in the posterior circulation. Among these, PCA trunk aneurysms are particularly rare and frequently present as dissecting aneurysms [1]. Surgical treatment of PCA aneurysms is often technically challenging because the operative field may be limited by temporal lobe retraction and the presence of the vein of Labbé. In addition, these aneurysms are frequently located near numerous perforating arteries, further increasing surgical complexity. Due to these anatomical constraints, an increasing number of studies have reported the effectiveness of endovascular treatment for PCA aneurysms [1,2]. Parent artery occlusion (PAO) is more frequently selected than reconstructive, parent artery-preserving strategies, particularly for distal PCA aneurysms. It has been associated with higher long-term complete occlusion rates [3,4]. However, postoperative complications, including hemianopsia and permanent hemiparesis due to cerebral infarction, are significantly more frequent in patients treated with PAO [3]. In particular, patients with a fetal-type posterior communicating artery (PCoA) are considered more likely to develop ischemic complications than those with an adult-type PCoA, possibly due to differences in the origin of perforating branches and the underdevelopment of collateral circulation [1]. In general, a fetal-type PCoA is defined as a configuration in which the PCA territory is predominantly supplied by the internal carotid artery (ICA) via the PCoA with a hypoplastic or absent P1 segment [5]. Even in aneurysms located distal to the P2 segment, a higher incidence of ischemic complications has been reported in patients with a fetal-type PCoA following PAO [6]. Taken together, the optimal treatment strategy in patients with fetal-type PCoA anatomy remains incompletely defined.

Here, we report a case of subarachnoid hemorrhage (SAH) caused by a ruptured aneurysm located in the distal P2 segment of the left PCA, treated with PAO via a fetal-type PCoA approach. Although successful prevention of rebleeding was achieved, postoperative ischemic complications occurred. This case is presented to suggest a possible increased risk of ischemic complications in PCA territories in the presence of fetal-type PCoA anatomy and highlight the importance of careful treatment selection, particularly when considering deconstructive strategies, such as PAO, in anatomically high-risk situations.

## Case presentation

**Figure 1:**
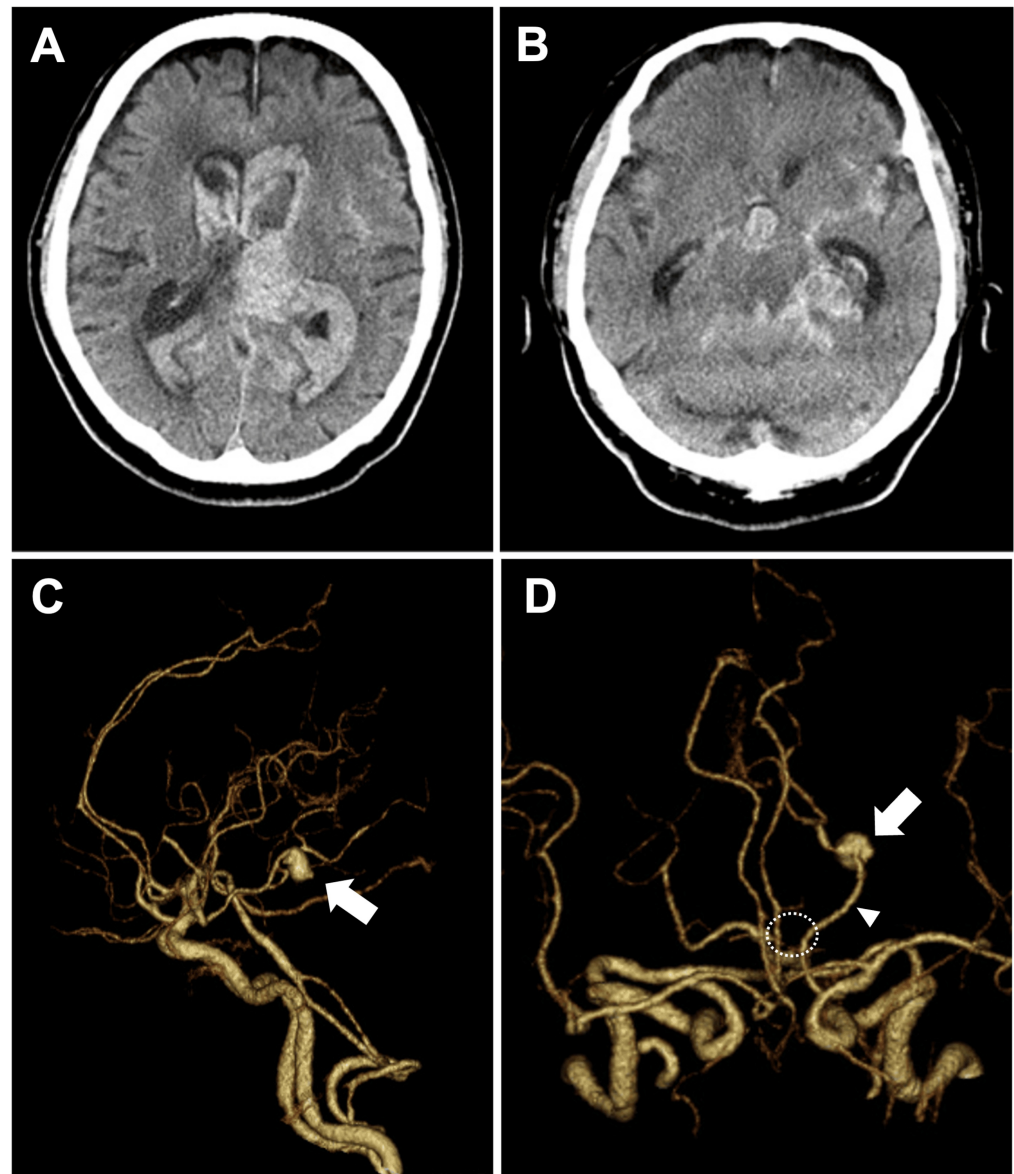
Initial CT findings of the ruptured posterior cerebral artery aneurysm (A, B) Axial non-contrast head CT images obtained on the day of onset show diffuse subarachnoid hemorrhage (SAH), with a particularly thick clot in the left ambient cistern. Acute obstructive hydrocephalus is also observed. (C) Lateral and (D) superior views of contrast-enhanced head CT demonstrate a ruptured aneurysm, considered the source of bleeding, located in the distal P2 segment of the left posterior cerebral artery (PCA) (white arrow). The left posterior communicating artery (PCoA) is the fetal-type (white arrowhead), and the ipsilateral P1 segment of the PCA is hypoplastic (dashed circle). CT: computed tomography

Our patient was a 76-year-old man with a history of hypertension and hyperuricemia who was transported to our hospital because of a sudden-onset severe headache and impaired consciousness. He arrived at our hospital within one hour of symptom onset. On arrival, his level of consciousness was E1V2M3 on the Glasgow Coma Scale (GCS), and he exhibited snoring respirations. Non-contrast brain CT revealed diffuse SAH with acute obstructive hydrocephalus. A particularly thick clot was observed in the left ambient cistern. Contrast-enhanced CT demonstrated an aneurysm located in the distal P2 segment of the left PCA. The ipsilateral PCoA was a fetal-type, and the P1 segment was hypoplastic (Figure 1).

He was diagnosed with SAH due to rupture of a left PCA aneurysm (Hunt and Kosnik grade V). Ventricular drainage was first performed to treat the acute hydrocephalus, followed by endovascular treatment. All procedures were performed on the day of onset.

**Figure 2:**
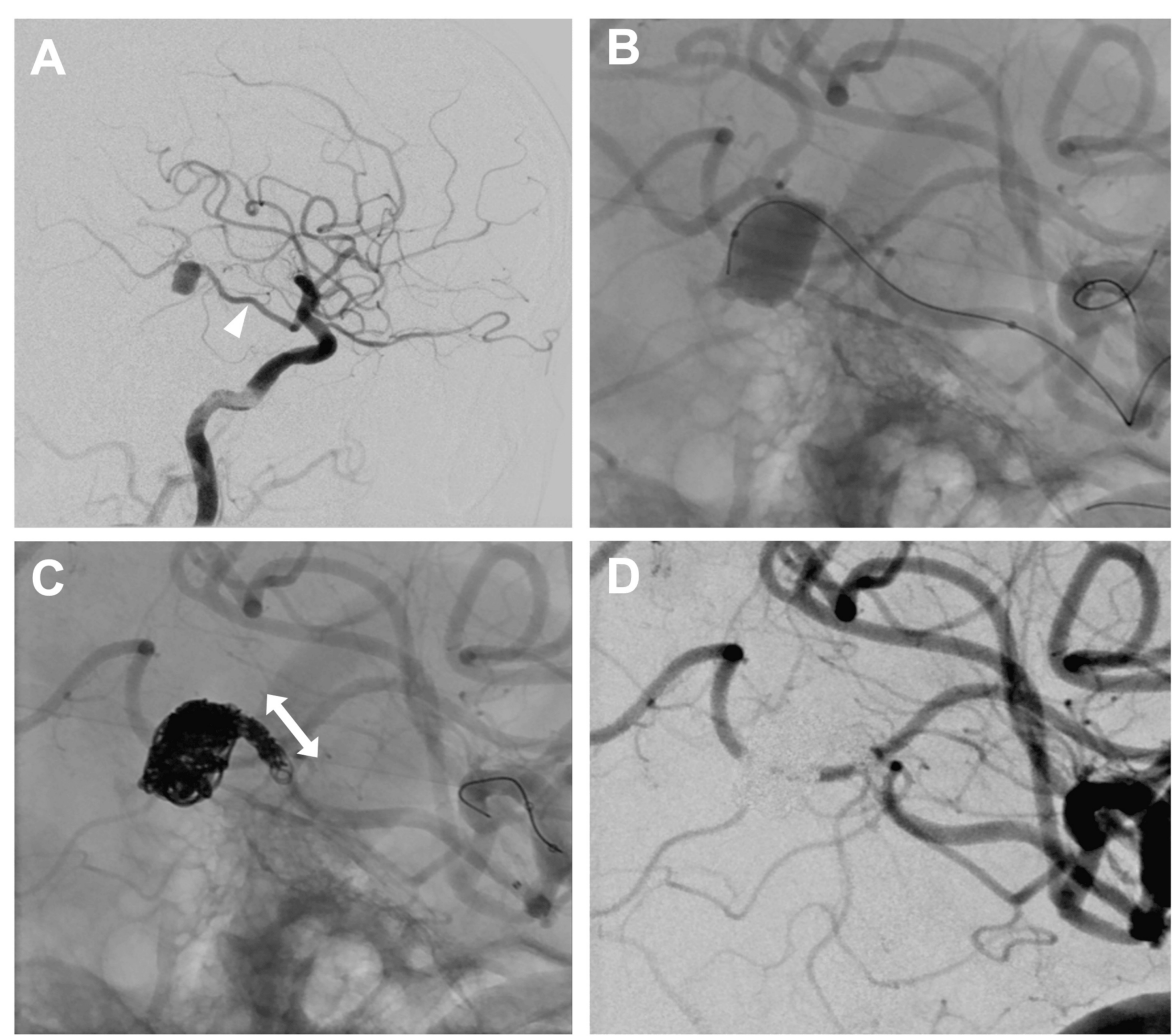
Endovascular treatment of ruptured PCA aneurysm via a fetal-type PCoA approach (A) Preoperative digital subtraction angiography (DSA), lateral view, demonstrates that the left PCA is supplied by the anterior circulation via a fetal-type PCoA (white arrowhead). (B, C) Lateral working-angle views show a 1.7-Fr microcatheter advanced into the aneurysm via the PCoA from the ipsilateral internal carotid artery. A total of 22 coils were deployed to achieve parent artery occlusion (PAO) of the aneurysm and the proximal parent vessel. The length of the occluded segment was approximately 5 mm (double-headed arrow). (D) Lateral working-angle view obtained immediately after the procedure demonstrates complete occlusion of the aneurysm and the parent artery. PCA: posterior cerebral artery; PCoA: posterior communicating artery

Immediately after ventricular drainage, endovascular treatment was initiated. Systemic heparinization was started during the procedure, and the activated clotting time (ACT) was maintained between 250 and 300 seconds. Digital subtraction angiography (DSA) revealed a ruptured aneurysm measuring 8.2 × 10.5 × 7.4 mm at the distal P2 segment of the left PCA. Although the aneurysm morphology was compatible with a saccular aneurysm, no branch vessels were observed near the aneurysm neck, and the possibility of a dissecting aneurysm could not be completely excluded. The ipsilateral P1 segment was hypoplastic, and the PCA was mainly supplied by the ICA through a fetal-type PCoA (Figures 2A, 2B). Because the ipsilateral P1 segment was hypoplastic, the lesion was approached via the PCoA from the anterior circulation. As the lesion involved the dominant hemisphere, we initially aimed to preserve the parent artery by performing selective coil embolization of the aneurysm whenever feasible. Reconstructive strategies, including stent-assisted techniques, were considered less suitable in the acute rupture setting due to the need for antiplatelet therapy. However, given the priority of preventing rebleeding, PAO was considered as a secondary strategy if complete aneurysm occlusion could not be achieved. An 8-Fr Optimo EPD balloon guiding catheter (Tokai Medical Products, Aichi, Japan) was introduced through the right femoral artery. To reduce microcatheter kickback during coil deployment, a 3.2/3.4-Fr distal access catheter (DAC; Guidepost; Tokai Medical Products, Aichi, Japan) was advanced into the PCoA, thereby providing additional support for a 1.7/2.4-Fr microcatheter (Excelsior SL-10; Stryker, Kalamazoo, MI, USA). Although selective aneurysm embolization was attempted, contrast filling within the aneurysm persisted, and complete occlusion could not be achieved. Therefore, the treatment strategy was changed to PAO. Advancing the DAC into the PCoA improved microcatheter stability, thereby enabling a short-segment, tightly packed PAO of approximately 5 mm in length. A total of 22 coils were deployed, and the procedure was completed after confirming the absence of contrast filling within the aneurysm (Figures 2C, 2D).

**Figure 3:**
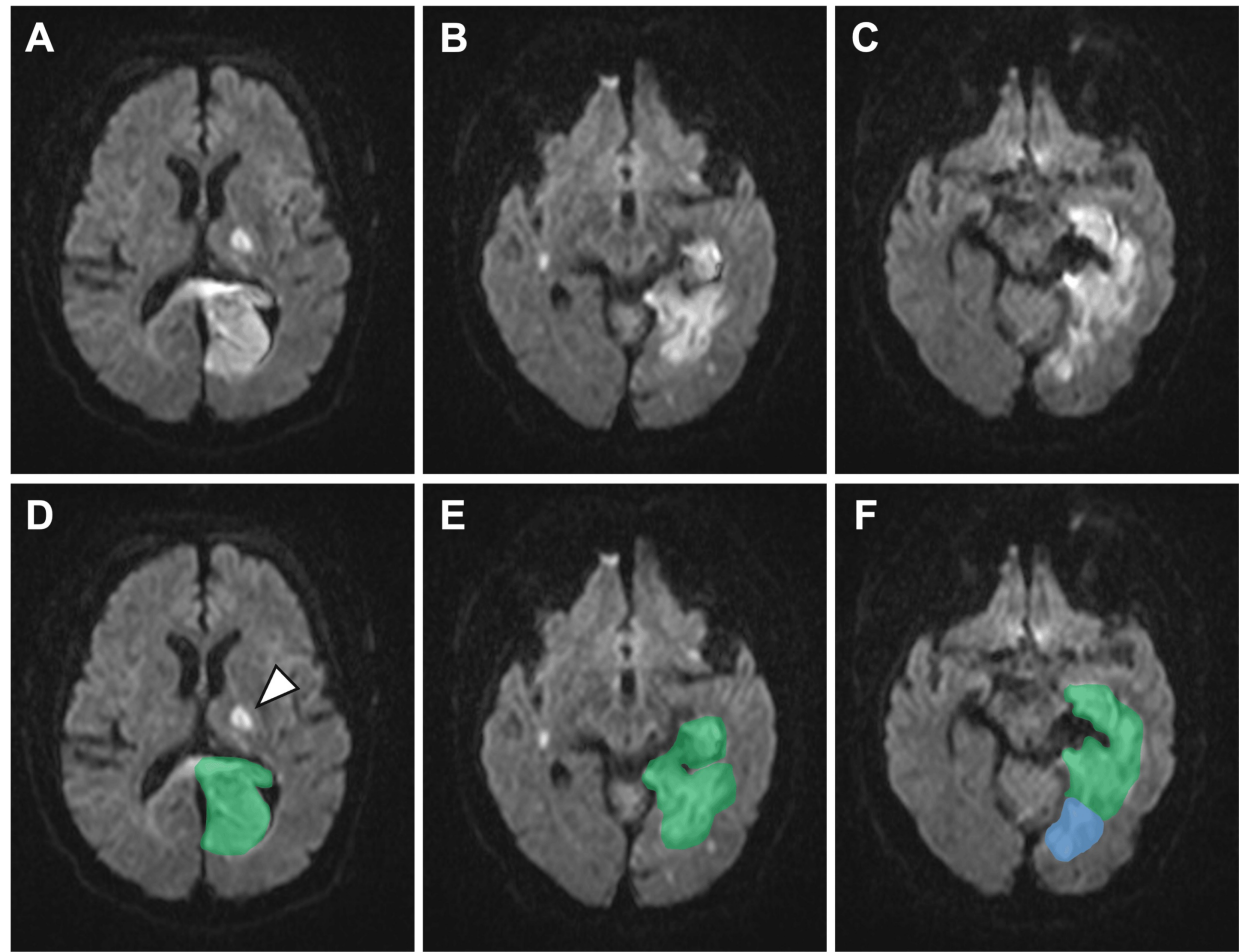
MRI on postoperative day 6 (A-C) Axial diffusion-weighted MRI obtained on postoperative day 6 demonstrates acute infarctions in the lateral thalamus within the thalamogeniculate artery territory and in the medial temporal and occipital lobes. (D-F) Territorial labeling of the infarctions corresponding to (A-C). The white arrowhead indicates infarction in the left lateral thalamus. The green-shaded region represents the medial temporal lobe, corresponding to a PCA-MCA border zone, and the blue-shaded region represents the occipital lobe, corresponding to the primary PCA territory. PCA: posterior cerebral artery; MCA: middle cerebral artery; MRI: magnetic resonance imaging

The level of consciousness improved to approximately E4V4M6 on the GCS by around postoperative day (POD) 5. However, MRI performed on POD 6 revealed diffusion restriction on diffusion-weighted imaging (DWI) in the ipsilateral lateral thalamus, as well as in the medial temporal and occipital lobes (Figure 3).

Occipital lobe infarction was considered a partially unavoidable consequence of PCA occlusion. In contrast, the lateral thalamic and medial temporal lobe infarctions in this case were likely attributable to hypoperfusion of the thalamogeniculate artery and temporal branches arising from the PCA, potentially influenced by the presence of a fetal-type PCoA. Intravenous fasudil hydrochloride and ozagrel sodium were administered continuously from POD 3 to POD 14. Given that the infarction was already extensive and well established, and considering the acute phase, antiplatelet therapy was not administered.

**Figure 4:**
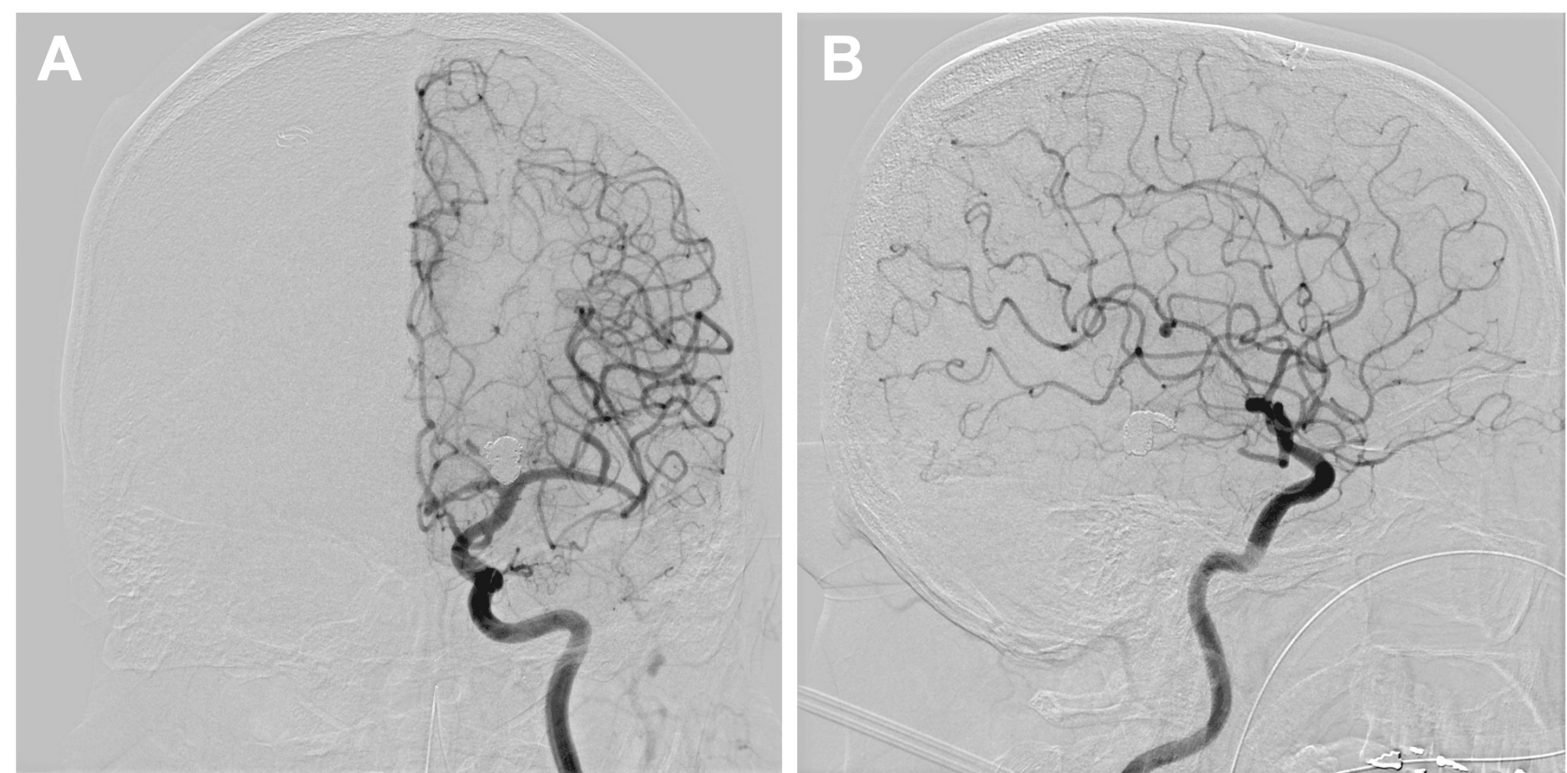
DSA on postoperative day 14 (A) Anteroposterior and (B) lateral views of DSA obtained on postoperative day 14 demonstrate complete occlusion of the aneurysm and the parent artery distal to the P2 segment, with no evidence of cerebral vasospasm. DSA: digital subtraction angiography

Follow-up DSA performed on POD 14 confirmed complete occlusion of both the aneurysm and the parent artery (Figure 4).

However, the patient developed right homonymous hemianopia, which was diagnosed by bedside confrontation visual field testing. He also exhibited higher cortical dysfunction, including attention deficits and memory impairment, as reflected by a Mini-Mental State Examination (MMSE) score of approximately 12, as assessed by a speech-language therapist. The cognitive impairment reflected by the MMSE score may be partly attributable to medial temporal lobe infarction. No motor deficits or cranial nerve abnormalities were observed. He was ultimately transferred to a rehabilitation hospital on POD 55 with a modified Rankin Scale (mRS) score of 4. The clinical course is summarized in Table 1.

**Table 1:** Clinical course timeline DSA: digital subtraction angiography; DWI: diffusion-weighted imaging; GCS: Glasgow Coma Scale; MRI: magnetic resonance imaging; mRS: modified Rankin Scale; PAO: parent artery occlusion; PCA: posterior cerebral artery; PCoA: posterior communicating artery; POD: postoperative day; SAH: subarachnoid hemorrhage

Time point	Clinical event	Findings/intervention
Day 0 (onset)	Sudden-onset headache	GCS E1V2M3; snoring respirations
Within one hour	Arrival at the hospital	Diagnosis: SAH due to rupture of a left PCA aneurysm with a fetal-type PCoA (Hunt and Kosnik grade V)
Day 0	Ventricular drainage	Performed for acute hydrocephalus
Day 0	Endovascular treatment	PAO performed
POD 6	MRI (DWI)	Infarctions in the lateral thalamus, medial temporal lobe, and occipital lobe
POD 3-14	Medical therapy	Fasudil hydrochloride and ozagrel sodium were administered
POD 14	Follow-up DSA	Complete occlusion of the aneurysm and the parent artery
POD 55	Outcome	Transferred to a rehabilitation hospital; mRS 4

## Discussion

In the present case, a ruptured PCA aneurysm with a fetal-type PCoA was treated by PAO, resulting in postoperative infarctions in the lateral thalamus, medial temporal lobe, and occipital lobe. Although PAO successfully prevented rebleeding, clinically significant neurological deficits occurred, including homonymous hemianopia and higher cortical dysfunction.

The observed infarction pattern may be explained by plausible anatomical mechanisms related to the vascular supply of the PCA territory. Thalamic infarction may be attributable to hypoperfusion of the thalamogeniculate artery arising from the P2 segment. In addition, medial temporal lobe infarction may reflect insufficient leptomeningeal anastomoses between the PCA and temporal branches of the middle cerebral artery (MCA). These mechanisms may have been influenced by the presence of a fetal-type PCoA.

However, these interpretations remain speculative. Other mechanisms cannot be excluded, and multiple factors may have contributed to the development of infarction in this case. Strict intraoperative ACT monitoring was maintained, and no thrombus formation was observed on serial intraoperative angiographic assessments. Nevertheless, the possibility of embolic complications cannot be completely excluded. In addition, although preoperative angiography confirmed that no obvious perforating branches were included within the occluded segment, involvement of perforators below the level of angiographic resolution cannot be ruled out. Furthermore, given the presence of high-grade SAH, SAH-associated factors, including vasospasm and microcirculatory impairment, may also have contributed to the development of infarction.

According to Zeal and Rhoton, the PCA can be divided into four segments (P1-P4), with P1 and P2 regarded as proximal segments and P3 and P4 as distal segments [7]. Approximately 80% of PCA aneurysms occur in the proximal segments, particularly in the P2 segment. Unlike aneurysms at other intracranial locations, saccular aneurysms at branching points are relatively uncommon in the PCA, and 80%-90% are reported to be dissecting aneurysms [1,8]. The PCA has rich collateral circulation that varies depending on the segment involved. Major collateral pathways include anastomoses between the P1 segment and branches of the superior cerebellar artery (SCA), between the P2 segment and the anterior choroidal artery (AchA) or temporal branches of the MCA, and between the P3-P4 segments and the posterior pericallosal artery arising from the anterior cerebral artery (ACA) [9]. Because important perforating arteries supplying the brainstem arise from the P1 segment, PAO at this level is generally not recommended due to the high risk of severe ischemic complications. In contrast, PAO for aneurysms distal to the P2 segment is considered relatively safe because of the presence of well-developed collateral circulation and a lower risk of ischemia [1,10]. However, in cases with a fetal-type PCoA, the P1 segment is frequently hypoplastic, and a greater number of perforating arteries may arise from the P2 segment. In addition, leptomeningeal anastomoses between the PCA and the MCA may be underdeveloped, potentially increasing the risk of temporal lobe ischemic complications after PAO [11]. Evaluation of collateral circulation based solely on DSA findings may be insufficient to determine the safety of PAO [9]. Balloon occlusion testing (BOT) has been reported as a useful method for the assessment of collateral circulation [12]. However, it is often impractical in patients with severe SAH, as in the present case. Therefore, careful preoperative assessment of aneurysm location and vascular anatomy, including PCoA configuration, is essential.

This case should be interpreted as an observational finding rather than definitive evidence of causation. The findings suggest a potential risk of ischemic complications following PAO, particularly in the presence of fetal-type PCoA anatomy. In this context, despite limitations related to the need for antiplatelet therapy, stent-assisted reconstructive treatment with preservation of the parent artery may be considered in selected cases as a potential alternative strategy. Therefore, treatment decisions should be individualized and approached with caution, especially in the acute rupture setting where therapeutic options may be limited.

## Conclusions

PAO is an effective treatment option for preventing rebleeding in ruptured PCA aneurysms, particularly when a dissecting aneurysm is suspected. However, the risk of postoperative ischemic complications may vary depending on vascular anatomy, especially in the presence of a fetal-type PCoA and the aneurysm location. Therefore, detailed preoperative vascular anatomical assessment and individualized treatment planning are essential. This case highlights the potential for increased ischemic risk associated with fetal-type PCoA anatomy following PAO and suggests the need for caution in such anatomical settings.
